# The COVID-19 Pandemic Can Impact Perinatal Mental Health and the Health of the Offspring

**DOI:** 10.3390/bs10110162

**Published:** 2020-10-23

**Authors:** Rafael A. Caparros-Gonzalez, Ana Ganho-Ávila, Alejandro de la Torre-Luque

**Affiliations:** 1 Department of Nursing, Faculty of Health Sciences, University of Granada, 18071 Granada, Spain; 2 Mind, Brain, and Behavior Research Center (CIMCYC), University of Granada, 18011 Granada, Spain; 3 Center for Research in Neuropsychology and Cognitive Behavioral Intervention, Faculty of Psychology and Educational Sciences, University of Coimbra, 3000-115 Coimbra, Portugal; ganhoavila@fpce.uc.pt; 4 Department of Legal Medicine, Psychiatry and Pathology, Universidad Complutense de Madrid, 28040 Madrid, Spain

**Keywords:** prenatal stress, antenatal stress, psychological stress, psychological well-bieng, physiological stress, cortisol, pregnancy, COVID-19, SARS-Co-2, offspring, neonate

## Abstract

The COVID-19 ongoing pandemic constitutes a major challenge for countries throughout the world due to the rapid spread of SARS-CoV-2 and devastating consequences in health. No one is free from COVID-19 impact. In this regard, pregnant women are not the exception. The COVID-19 outbreak represents a massive source of stressful agents for women and their babies during the perinatal period. The COVID-19 pandemic has been suggested to potentially have short- and long-term detrimental effects on pregnant women and the baby. These adverse consequences range from mental to medical diseases. During the last centuries, several dreadful and fatal incidents have put pregnant women and their babies at higher risk of mortality and health deterioration. For example, it has been informed that women exposed to the 1918 flu pandemic (commonly known as the Spanish flu) while pregnant showed higher rates of premature delivery in the short term. Long-term consequences have also been reported and individuals (both males and females) who were exposed to the 1918 flu pandemic while in utero had a higher risk of developing schizophrenia, diabetes, coronary heart disease or cancer throughout their lifespan.

The ongoing COVID-19 pandemic constitutes a major challenge for countries throughout the world due to the rapid spread of SARS-CoV-2 and the devastating consequences for health. No one is free from the impact of COVID-19. In this regard, pregnant women are not an exception. The COVID-19 outbreak represents a massive source of stress for women and their babies during the perinatal period. The COVID-19 pandemic has been suggested to potentially have short- and long-term detrimental effects on pregnant women and their babies. These adverse consequences range from mental illnesses to medical diseases. During the last few centuries, several dreadful and fatal incidents have put pregnant women and their babies at higher risk of mortality and health deterioration. For example, it has been noted that women exposed to the 1918 flu pandemic (commonly known as the Spanish flu) while pregnant showed higher rates of premature delivery in the short term. Long-term consequences have also been reported and individuals (both males and females) who were exposed to the 1918 flu pandemic while in utero had a higher risk of developing schizophrenia, diabetes, coronary heart disease or cancer throughout their lifespan [1,2].

The environment surrounding pregnant women affects the developing fetus while still in the womb. In fact, this environment imprints and programs the health and disease of the offspring from birth to the end of life. This hypothesis was posited by David Barker and is known as the Developmental Origin of Health and Disease (DOHaD) hypothesis [3]. An evolutionary perspective suggests that an environment with high levels of stress can prepare the developing fetus to adapt to the extreme circumstances that the child will be exposed to after his/her birth. In this regard, attention deficit hyperactivity disorder, for instance, has been associated with higher appraisals of danger while anxiety disorders have been related with higher vigilance [4].

During the ongoing global pandemic of COVID-19, potential agents of maternal stress have been described so far, and all of them may lead to perinatal mental health issues. Some of these agents may come directly from the virus and adjustment to related disease, whereas others are indirectly related to SARS-CoV-2. In order to limit the SARS-CoV-2 spread, worldwide governments have imposed some behavioral adjustments, such as country lockdown, social distancing and mobility restriction. Social distancing represents a crucial approach that may lead to adverse mental health effects. Humans are instinctively social and tend to gather as a mechanism to cope with high levels of stress and a way to increase resilience [5]. Isolation and limitation of movement may result in pregnant women experiencing a lack of social support from friends, relatives and partners, economic difficulties, remote work and potentially staying in overcrowded homes. Further stressors related to social distancing during the COVID-19 pandemic for women during the perinatal period are the challenges of having to school children at home, additionally woman are facing increased risk related to intimate partner violence since during the lockdown women had to cohabit with a potential aggressor, reduced number of antenatal and postnatal appointments, restrictions related to partner’s participation during birth and changes associated with breastfeeding recommendations [6,7].

A growing body of evidence appears to support the detrimental psychological effects the COVID-19 pandemic and related policies can have on pregnant women. Worldwide, researchers are moving forward to understand the extent to which these catastrophic circumstances can shape perinatal mental health and how high levels of stress suffered by pregnant women can program the well-being of the offspring. However, a major effort should be undertaken to understand mental health consequences and to improve optimal mental health service provision. In this respect, the absence of psychometrically validated psychological measures to assess pandemic-related stress among pregnant women has resulted in the development of new high-standard and cross-cultural measures. These measures will help researchers gain new insights into the assessment of psychological stress during the COVID-19 outbreak and potential upcoming pandemics (e.g., [8,9]). So far, high levels of anxiety and depression have been found in pregnant women from a variety of geographical and cultural backgrounds, such as Canada, UK, Australia, India and China [10,11]. Pregnant women from Israel were reported to have high levels of COVID-19 anxiety, and Arab women displayed the highest levels of psychological stress when compared with Jewish pregnant women [12]. Additional inequalities among certain groups of pregnant women have also been discovered, which highlights the need to include women in high-risk groups. A national cohort study developed in the UK found that women over 35 years old, overweight/obese and those from a minority ethnicity might have a higher risk of SAR-CoV-2 infection. Vulnerable pregnant women are among those who have higher levels of stress related to worries about getting infected or spreading SARS-CoV-2 [13,14]. Further wide-reaching projects focusing on the psychological impact of the COVID-19 pandemic on pregnancy are ongoing, such as the COVID-19 and Perinatal Experiences Study (COPE) [15].

To sum up, pregnant women and their offspring have become high-risk segments of the population in this unprecedented time [16]. Policymakers and health care professionals have made a major effort to tackle the impact of COVID-19. Despite this effort, preventive programs and interventions should be implemented to ameliorate the mental health burden from COVID-19. In addition, it should be compulsory for every pregnant woman to be assessed for mental health issues at every stage of her pregnancy and postpartum [6]. This tailored assessment could help develop adjustment strategies to cope with the pandemic and reduce the probability of certain diseases being developed in the offspring later in life. Finally, a call for further research on maternal mental health deserves to be made. Researchers are encouraged to focus their efforts on identifying protective and risk factors and to develop interventions for maternal mental health during the COVID-19 pandemic.
